# Spatial variation in anuran richness, diversity, and abundance across montane wetland habitat in Volcanoes National Park, Rwanda

**DOI:** 10.1002/ece3.5054

**Published:** 2019-03-13

**Authors:** Yntze van der Hoek, Deogratias Tuyisingize, Winnie Eckardt, Nuria Garriga, Mia A. Derhé

**Affiliations:** ^1^ The Dian Fossey Gorilla Fund International Musanze Rwanda; ^2^ Department of Evolutive Biology, Ecology, and Environmental Sciences University of Barcelona Barcelona Spain

**Keywords:** community composition, elevational gradient, frogs, species‐area relationship, wetland desiccation

## Abstract

The spatial distribution of species has long sparked interest among ecologists and biogeographers, increasingly so in studies of species responses to climate change. However, field studies on spatial patterns of distribution, useful to inform conservation actions at local scales, are still lacking for many regions, especially the tropics. We studied elevational trends and species‐area relationships among anurans in wetland habitats within Volcanoes National Park (VNP) in Rwanda, part of the biodiverse Albertine Rift region. In VNP, wetlands are key sites for anuran reproduction, and anurans are likely threatened by wetland desiccation which has occurred for the last few decades. Between 2012 and 2017, we sampled anuran communities in ten VNP wetlands located along an elevational gradient of c. 600 m (from 2,546 to 3,188 m a.s.l.) and found at least eight species, including at least two Albertine Rift Endemics. We show that species richness, diversity, and abundance likely decline with a decrease in wetland size and with an increase in elevation, though additional sampling (e.g., at night) might be needed to derive definite conclusions. Larger wetlands at lower elevations contained most species and individuals, which indicates the potential threat of wetland size reduction (through desiccation) for anuran conservation. However, we also found that wetlands differed in species composition and that some species (e.g., *Sclerophrys kisoloensis*) were likely restricted in distribution to only a few of the smaller wetlands—suggesting that the conservation of each individual wetland should be prioritized, regardless of size. We propose that all wetlands in VNP require additional conservation measures, which should be based on knowledge gathered through long‐term monitoring of anuran communities and research on drivers of wetland decline. Only such extended research will allow us to understand the response of anurans in VNP to threats such as climate change and wetland desiccation.

## 1 INTRODUCTION

An understanding of the spatial distribution of biodiversity allows us to address how evolutionary and ecological processes shape ecosystems and guides conservation efforts, such as the spatial planning of protected areas. Furthermore, only with an understanding of current spatial patterns will we be able to assess species responses to environmental change (e.g., climate or land cover change) or predict future distributions of species under different scenarios of change. Patterns of elevational variation in species richness and species‐area relationships are two especially well‐studied topics in biogeography, enabling us to increasingly understand how historic, evolutionary, climatic, and geometric factors are linked to the distribution of biota (Dengler, 2009; Laiolo, Pato, & Obeso, 2018; Lomolino, 2001).

In certain regions, and for some taxa, species richness peaks at low elevations or plateaus at mid elevations due to geometric constraint known as the mid‐domain effect (Colwell, Rahbek, & Gotelli, 2004). Alternatively, richness may decline monotonically with an increase in elevation, a pattern that closely resembles latitudinal trends and is considered to be predominantly driven by environmental correlates of elevation, such as a decline in temperature (Rahbek, 1995). Which pattern dominates in a given ecosystem or locality depends on many factors, such as the scale of study (Rahbek, 2005) and local and regional climatic conditions (Fu et al., 2006). In fact, elevational patterns may even differ between southern and northern slopes of the same mountain (Shuai, Ren, Yan, Song, & Zeng, 2017). Thus, despite the wealth of existent literature, we cannot extrapolate from one study to the next. It, therefore, remains imperative to extend studies of elevational species distribution to fragile or threatened ecosystems in understudied regions, such as Central and East Africa, where these studies may inform conservation at local scales.

Similar to the exploration of elevational patterns, we have advanced considerably in our understanding of the relationships between species diversity and area; relationships that were first given context by the theory of island biogeography (MacArthur & Wilson, 1967). Although a general tendency of increasing species richness with increases in area of investigation remains a common result of studies in biogeography and landscape ecology, there are now many alternative explanations for underlying mechanisms (for an overview of some of the most important, long‐standing, hypotheses see McGuinness (1984) and Connor and McCoy (1979)). Given this complexity of underlying mechanisms and associated describing models, it is unsurprising that it is hard to generalize species‐area relationships, even within one taxon, such as amphibians.

Anurans and other amphibians play many intrinsic roles in ecosystems (Hocking & Babbitt, 2014), and local extirpation of amphibians may lead to severe alterations of ecosystem structure and functioning (Whiles et al., 2006). Unfortunately, many amphibian populations are experiencing declines because of a host of interacting threats, including climate change, chemical contamination, disease, the spread of invasive species, and habitat loss and degradation (Blaustein et al., 2011; Kiesecker, Blaustein, & Belden, 2001). As a result, amphibians are experiencing a global crisis of population decline and extirpation (Stuart et al., 2004; Wake & Vredenburg, 2008), and many Afrotropical species are equally threatened (Hillers, Veith, & Rödel, 2008; Zancolli, Steffan‐Dewenter, & Rödel, 2014).

Here, we explore the existence of elevational gradients and species‐area relationships in species richness, diversity, and abundance among anurans (i.e., frogs and toads) in the Virunga Massif, which is part of the Albertine Rift biodiversity hotspot in Central Africa. We know relatively little of anuran ecology in the Virunga Massif, despite our recognition of the wealth of biodiversity, high levels of endemism, and considerable conservation challenges in the wider region (Plumptre et al., 2007). However, we do know that amphibians of the Virunga Massif are likely threatened by climate change and habitat loss, degradation, and fragmentation (Plumptre et al., 2007; Salerno et al., 2017). We can also predict that anuran distribution in the Virunga Massif is at least partially determined by elevational gradients, as is the case in nearby regions in East Africa (Malonza & Veith, 2011; Poynton, Loader, Sherratt, & Clarke, 2006; Zancolli et al., 2014). And finally, we predict to find positive effects of habitat size on anuran species richness, diversity, and abundance, as has been shown for other anuran communities in the tropics (Almeida‐Gomes, Vieira, Rocha, Metzger, & Coster, 2016).

Volcanoes National Park (VNP) in Rwanda, which protects a part of the Virunga Massif, experiences enormous anthropogenic pressures. Most impacts stem from a particularly high human population density near the park (Bush, Ikirezi, Daconto, Gray, & Fawcett, 2010). The volcanic soil found in and near the park allows for highly productive agriculture, which drives local people to cultivate up to the edge of the park (Weber, 1987). In turn, direct water extraction from the park, alterations of ground water levels through agriculture activities, widespread use of nonnative plant species that may affect hydrological regimes (e.g., *Eucalyptus *spp.; Dye, 2013), and altered frequencies of rainfall (Campos et al., 2017), possibly contributed to observed reductions of water availability inside wetlands (The Dian Fossey Gorilla Fund International, unpublished data). Over the last decades, previously permanently wet ponds in certain wetlands now remain dry for parts of the year, while other wetlands have dried up completely and are experiencing associated vegetational succession toward shrubbery and forest. This process of wetland desiccation is likely to have a negative impact on the anurans particular to this habitat (McMenamin, Hadly, & Wright, 2008), especially in combination with changes in climatic factors, such as altered frequency and periodicity of rainfall (Hartter et al., 2012).

Previous studies in VNP showed the presence of at least nine different anuran species (Roelke & Smith, 2016). Many, if not all, of these species have some association with wetland habitat, especially with regards to reproduction. Of these species, the Karisimbi tree frog *Leptopelis karissimbensis* is currently considered Vulnerable (IUCN SSC Amphibian Specialist Group, 2016). We investigated the distribution of these species in wetlands of varying sizes, found along an elevational gradient of c. 600 m that spans all vegetation zones in VNP. In contrast to previous studies conducted in the Albertine Rift region, which address broad‐scale distribution patterns across vegetation types (Behangana, Kasoma, & Luiselli, 2009; Malonza & Veith, 2011; Zancolli et al., 2014), we focus on fine‐scale patterns within broadly similar vegetation types (wetlands). In particular, we provide an overview of the anuran community composition of each wetland and test for the separate effects of elevation and wetland size on anuran distribution. This information provides a baseline to test what drives anuran distribution and anuran responses to increasing threats in this biodiverse, but fragile, region of Central Africa.

## 2 METHODS

### 2.1 Data collection and study area

**Figure 1:**
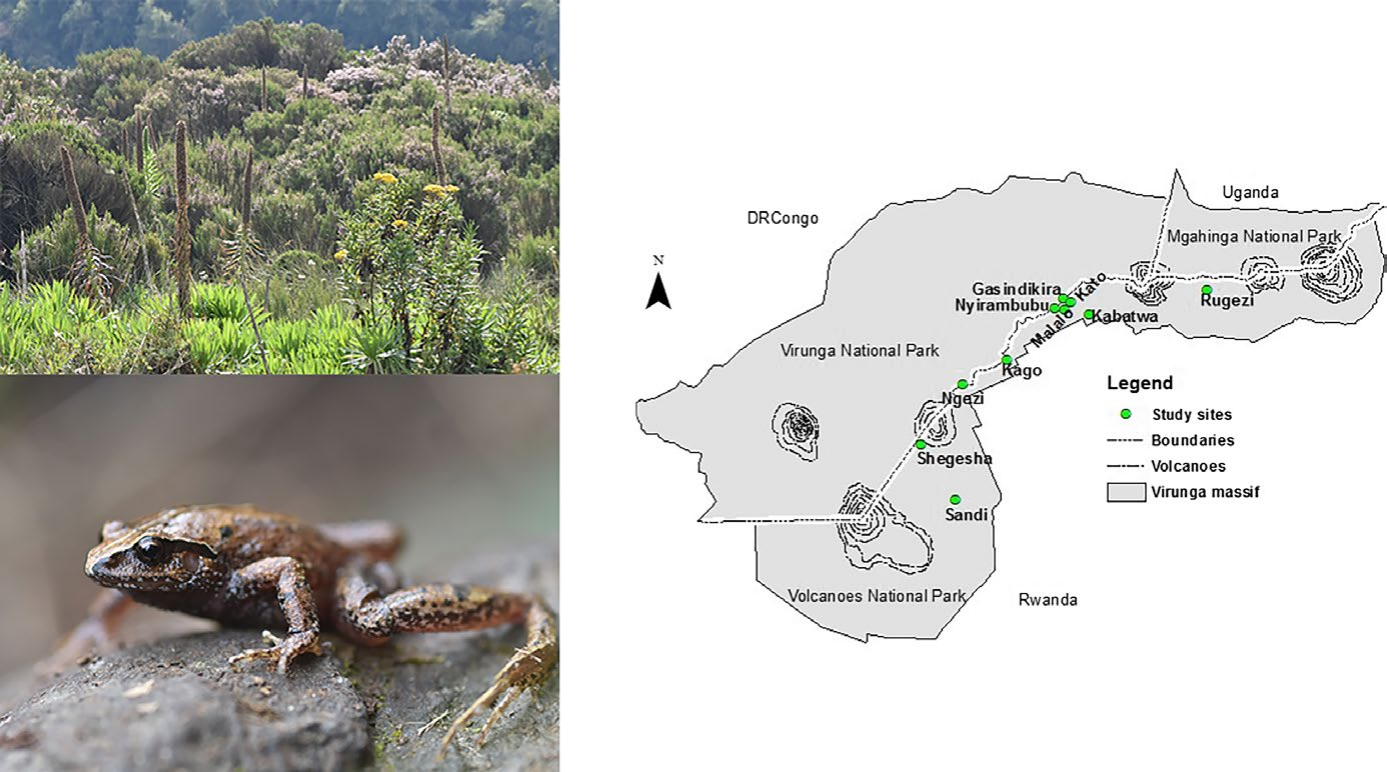
Location of focal wetlands (example top left photo) in which we sampled anurans (example *Leptopelis karissimbensis* bottom left) within Volcanoes National Park, Rwanda. The map shows the boundaries of the park as well as the adjacent Virunga National Park (Democratic Republic of Congo) and Mgahinga National Park (Uganda), which together protect a large part of the Virunga Massif

Data collection was undertaken by the biodiversity team of the Dian Fossey Gorilla Fund's Karisoke Research Center. Between 2012 and 2017, we sampled anurans in ten wetlands within Volcanoes National Park (VNP), Rwanda (1°21'–1°35'S, 29°22'–29°44'E; Figure 1). There are two distinct rainy seasons in VNP (March to May and September to November), and two dry seasons (June to August and December to February). Detailed published climatic data are only limitedly available for VNP, but mean annual rainfall reaches approximately 2,200 mm, average daily temperatures range from approximately 9°C at lower elevations to 0°C at higher elevations, and monthly variation in rainfall can be substantial (Campos et al., 2017).

The vegetation in VNP includes mixed forest, bamboo, *Hagenia* woodland, herbaceous, brush ridge, subalpine and alpine zones (Plumptre, 1991), whereas the vegetation inside the focal wetlands is characterized by species such as *Juncus oxycarpus* and *Mariscus karisimbiensis*, with encroachment by the dryland species *Hypericum revolutum* and *Yushania alpina* at many of the study sites. Focal wetlands ranged in size from approximately 99 to 45,355 m^2^ and were found between 2,546 and 3,188 m.a.s.l. (Table 1).

**Table 1:** Characteristics of focal wetlands in Volcanoes National Park, Rwanda

Wetland	Elevation (in m.a.s.l.)	Size (in m^2^)	Waterbody type	Seasonality of waterbody
Kabatwa	2,546	99	Pond	Seasonal
Kago	2,578	983	Marsh	Permanent
Rugezi	2,583	44,882	Stream	Permanent
Malalo	2,648	27,000	Swamp	Permanent
Nyirambubu	2,655	19,700	Marsh	Permanent
Kato	2,672	1,550	Pond	Seasonal
Gasindikira	2,706	8,750	Marsh	Permanent
Ngezi	2,861	28,850	Bog	Permanent
Shegesha	3,091	45,355	Marsh	Permanent
Sandi	3,188	5,725	Pond	Seasonal

Every study year, we visited each wetland two to five times between November and March and searched for anurans using the Visual Encounter Survey method (Crump & Scott, 1994) while walking 5 m wide strip transects that were spaced 15 m apart. During each visit, three or four observers slowly walked each transect parallel to each other, gently separating vegetation (e.g., clusters of grass) and looking at all sides of leaves. We did not monitor waterbodies for tadpoles, juveniles or adults, and though we used vocal cues to guide us to the location of individuals we did not abandon our gradual search method to record calling individuals (instead recording them as we reached them). The length and number of transects were consistent for each wetland across years but differed between wetlands to account for wetland size. To adjust for these varying sampling efforts across wetlands, we opted to use “site visits” as the sampling units in further statistical analyses, while sampled area (as a proxy of sampling effort) was used as an offset in our models (see below). Finally, we note that we were obliged to conduct all visits during daylight hours, despite the potential underestimates of abundance and richness this may have generated, because of restrictions on evening use of the park.

Unfortunately, we were unable to collect tissues and adopt molecular techniques for specimen identification. That said we did aim to collect photographic evidence to maintain a base of material for further clarification of identities (e.g., by consulting external experts). For the first three years of our study, we aimed to photograph the dorsal side of each specimen, a method we had to abandon in 2015 due to logistic restrictions. We thereafter aimed to photograph at least one specimen of each species found during every visit to a wetland. Despite these efforts, we acknowledge that both confusing taxonomy and morphological similarity between various congeneric species may have introduced some inaccuracy regarding the identities of certain specimen, see Discussion. For taxonomy, we followed Frost (2018), whereas we used information from for example, Zimkus, Rodel, and Hillers (2010), Roelke, Greenbaum, Kusamba, Aristote, and Smith (2011), and Channing, Dehling, Lotters, and Ernst (2016) to confirm that our species records were plausible in terms of geographic distribution.

### 2.2 Statistical analyses

To estimate species richness for each wetland and to assess sampling efficiency, we generated sample‐based observed species richness rarefaction curves with 95% confidence intervals, using the “vegan” (Oksanen et al., 2007) package in R (R Core Team, 2014). For this, we used the accumulative data for all years and site visits. In addition, we calculated estimated species richness as the mean of four abundance‐based species richness estimators (CHAO, JACK1, JACK2 and Bootstrap), following 999 randomizations of observed species richness. We subsequently calculated sampling completeness as the ratio between observed and estimated species richness. We estimated species diversity using the Shannon–Wiener index, which takes species richness and the relative proportions of species found in each wetland into account.

After testing for potential collinearity among variables using Pearson's correlations, and finding none, we tested for the effects of wetland size using generalized linear mixed effects models (glmm) constructed with the “lme4” package (Bates, Maechler, Bolker, & Walker, 2015) for models with abundance and species richness as response variables. We used “glmmTMB” (Brooks et al., 2017) for zero‐inflated glmms with Shannon–Wiener diversity as response variable. In these models, we treated wetland size as a fixed effect, sampling area (as a proxy of sampling effort) and elevation as offsets, and survey year and wetland identity as random effects. Next, we looked at the separate effect of elevation, for which we constructed similar models with abundance, species richness and Shannon–Wiener diversity as response variables, but with elevation as a fixed effect, survey year and site identity as random effect terms, and sampling area and wetland size as offsets. Appropriate error structures were applied for all models, and we used Chi‐square to compare models to null models.

To assess the similarity of wetlands in terms of their species composition, we used a nonmetric multidimensional scaling (NMDS) ordination analysis using Bray‐Curtis pairwise distances based on standardized, square‐root‐transformed abundance data (to reduce the influence of the most dominant species). We visualized results of this analysis in an ordination plot and used a permutational multivariate analysis of variance (*adonis* in the “vegan” package) to test for differences in Bray‐Curtis similarity between wetlands.

## 3 RESULTS

**Figure 2:**
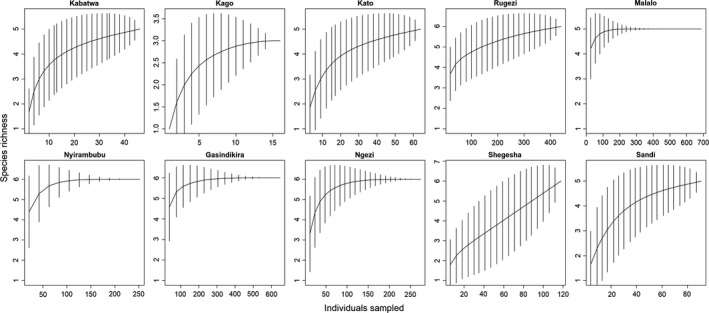
Species accumulation curves constructed using sample‐based rarefaction curves for ten focal wetland sites

Between 2012 and 2017, we recorded 2,638 specimens of at least eight anuran species (Tables 2 and 3), of which at least two are Albertine Rift Endemics (ARE). The most abundant species was *Hyperolius castaneus *(ARE), which was found at all sites, whereas we recorded only two specimens of *Sclerophrys kisoloensis*. Given the morphological similarity between *Leptopelis karissimbensis *(ARE) and *L. kivuensis *(ARE), we acknowledge that certain individuals that we identified as *L. karissimbensis *in the field could actually be *L. kivuensis*, and as such we opted use *L. karissimbensis/kivuensis *for further analyses. In addition, although we are certain that we have recorded both *Phrynobatrachus graueri *and *P. parvulus*, it was difficult to identify certain specimen of *Phrynobatrachus *to species‐level in the field. We thus also merged these two species as *P. graueri*/*parvulus *for further analyses. For reference, a list of all species records can be found in Supporting Information Appendix S1, and we added photographs of each species in Supporting Information Appendix S2, both found in Supporting Information. Species accumulation curves and species richness estimators suggest that additional sampling, at least during the day and during the time of the year of our annual sampling (see Discussion), would not have yielded many additional species for most wetlands (ratio observed/estimated richness >0.83) except for the wetland known as Shegesha, where some additional species might be found with additional sampling (Table 2, Figure 2).

**Table 2:** Summary of anuran sampling in ten focal wetlands in Volcanoes National Park, Rwanda, between 2012 and 2017. Given are the total abundance relative abundance (per hectare sampled), observed (Sobs) and estimated (Sest) species richness (± *SD*), and proportion of estimated species detected (Sobs/ Sest) in each wetland.

Wetland	Abundance	Relative abundance	Sobs	Sest	Sobs/Sest
Kabatwa	46	306.7	5^a^	5.72 ± 0.3	0.87
Kago	16	31.4	3	3.03 ± 0.1	0.99
Rugezi	440	115.8	7^a^	6.72 ± 0.3	1
Nyirambubu	252	105.0	6^a^	5.00 ± 0.0	1
Malalo	689	149.8	5^a^	6.03 ± 0.1	0.83
Kato	64	50.8	5^a^	5.58 ± 0.5	0.90
Gasindikira	648	231.4	6	6.6 ± 0.5	0.91
Ngezi	274	171.3	7^a^	6.01 ± 0.0	1
Shegesha	118	84.3	6^a^	9.68 ± 1.7	0.62
Sandi	91	60.7	5^a^	5.6 ± 0.5	0.89

a During field work, we identified that both *P. graueri *and *P. parvulus* are likely to be present in eight wetlands, thus there is likely at least one more species in these wetlands than presented here.

**Table 3:** Anuran species and number of specimens found in each focal wetland in Volcanoes National Park, Rwanda, between 2012 and 2017

Species	Kabatwa	Kago	Rugezi	Nyirambubu	Malalo	Kato	Gasindikira	Ngezi	Shegesha	Sandi	Total	Number of wetlands
*Amietia nutti*	—	2	3	11	—	—	18	4	1	—	39	6
*Sclerophrys kisoloensis*	—	—	1	—	—	—	—	—	—	1	2	2
*Hyperolius castaneus *(ARE)	22	5	260	150	183	43	516	199	16	8	1,402	10
*Hyperolius cinnamomeoventris*	1	—	17	19	13	3	46	26	1	3	129	9
*Hyperolius viridiflavus*	3	—	—	57	44	1	26	16	1	—	148	7
*Leptopelis karissimbensis/kivuensis *(ARE)^a^	13	9	65	10	184	9	37	22	1	4	354	10
*Phrynobatrachus graueri/parvulus*	7	—	94	5	265	7	5	7	98	85	278	9
Total abundance	46	16	440	252	689	64	648	274	118	91	2,638	
Number of species	5^b^	3	7^b^	6^b^	5^b^	5^b^	6	7^b^	6^b^	5^b^		

Note

ARE: Albertine Rift Endemic.

a Both *Leptopelis karissimbensis and L. kivuensis *are AREs.

b During field work, we identified that both *P. graueri *and *P. parvulus* are likely to be present in eight wetlands, thus there is likely at least one more species in these wetlands than presented here.

**Figure 3:**
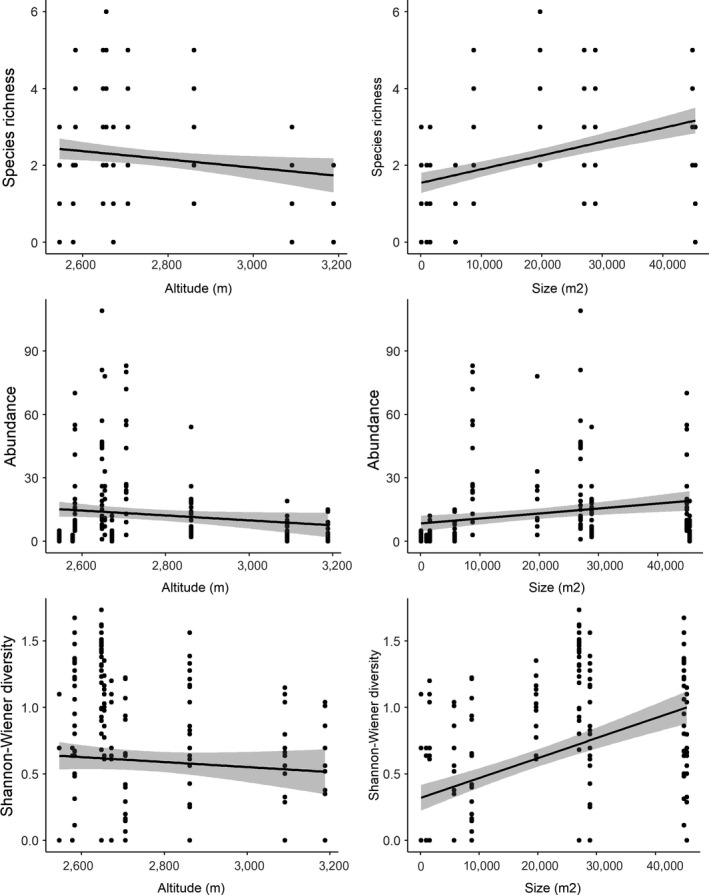
Effects of elevation and wetland size on anuran abundance, species richness and Shannon–Wiener diversity, showing a linear regression (black line) ± *SE* (gray shade)

**Figure 4:**
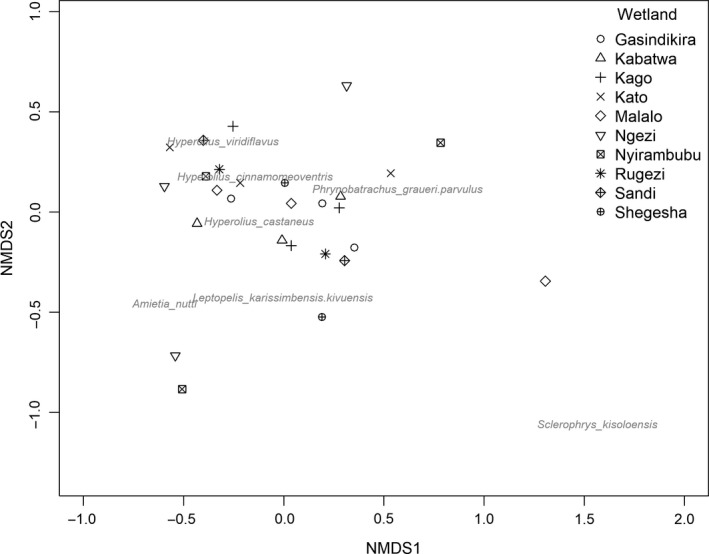
Nonmetric multidimensional scaling (NMDS) ordination of anuran community assemblages at different focal wetland sites, based on square‐root transformed, standardized abundance data (stress 0.14). Each point represents accumulative data for the 2012–2017 study period for a single transect, and the number of transects (i.e., points) ranges between one and four for different focal wetland sites

Elevation had a significant negative effect on anuran richness (χ^2^ = 9.94, *df* = 1, *p = *0.002), abundance (χ^2^ = 11.01, *df* = 1, *p < *0.001), and Shannon–Wiener diversity (χ^2^ = 9.28, *df* = 1, *p = *0.002; Figure 3). Wetland size had a significant positive effect on amphibian species richness (χ^2^ = 7.95, *df* = 1, *p* = 0.005), abundance (χ^2^ = 4.26, *df* = 1, *p* = 0.039), and Shannon–Wiener diversity (χ^2^ = 9.14, *df* = 1, *p = *0.003). In addition, species composition differed between wetlands though not necessarily following an elevational gradient. The NMDS ordination, which showed a good representation of wetland dissimilarity (stress value = 0.14; Clarke, 1993), indicates that wetland sites fail to form distinct clusters—though for example *Hyperolius *species seem to occur at relatively similar locations (Figure 4). Although anuran species composition often differed more within wetland (between transects) than between wetlands, statistical analyses did indicate that species composition differed significantly between wetlands (*adonis* method: *R* ^2^ = 0.84, *df* = 9, *p < *0.001).

## 4 DISCUSSION

Although we urge that our results should be interpreted with caution, due to biases introduced by skewed sampling efforts, such as a lack of nighttime sampling (see below for further discussion), we emphasize that there is considerable spatial variation among the anuran communities of the study wetlands in VNP. We found most diverse and abundant anuran communities at low elevations and in large wetlands, which is important information for park management. Wetlands at lower elevations are closer to park boundaries, and thus potentially more vulnerable to human disturbance. Additionally, ongoing desiccation is causing a reduction in the size of wetlands (The Dian Fossey Gorilla Fund International, unpublished data), which in turn might lead to the loss of species or reductions in population sizes. However, the drivers of both elevational and area effects on anuran communities in VNP require further investigation, as do the causes and extents of threats to the wetlands.

Most studies of elevational gradients in anuran distribution, including those conducted in the Albertine Rift region (Behangana et al., 2009), focus on broad‐scale trends. Usually, this implies that species are sampled from a range of habitat types, and that the effects of temperature, rainfall, and other correlates of elevation are conflated with a turn‐over in habitat type. As pointed out by Sinsch, Luemkemann, Rosar, Schwarz, and Dehling (2012), patterns in anuran diversity with elevation are typically associated with abrupt declines when moving from lowlands to mid elevations, and then again when going from mid elevations to Afromontane communities. For example, Behangana et al. (2009) showed that amphibian communities in the wider Albertine Rift region are most diverse in mature rainforests with dense canopy covers at lower elevations, and less so in more open habitats found at higher elevations. Here, we confirm that anuran richness, diversity, and abundance are likely to decline with elevation in the wetlands of VNP, which shows that these trends exist even within one relatively homogeneous habitat type (wetlands) across a relatively small elevational range. Anuran distributions are thus not only determined by a turn‐over between habitats, but potentially also by the effects of elevation, a proxy for climatic differences, per se.

We did not test for geographic and mid‐domain effects on the elevational distribution of anurans as we focused on fine‐scale trends among populations sampled across a relatively short gradient (~600 m) at mid‐to‐high elevations (i.e., we had “truncated sampling,” see McCain & Grytnes, 2010). Perhaps, when assessed over a larger elevational gradient, overall anuran richness may reach a plateau at mid elevations, although previous studies in the Albertine Rift suggest that it is more likely that amphibians continuously decline in richness and abundance with an increase in elevation (Behangana et al., 2009).

Few studies have shown patterns of decline in anuran abundance within species across elevational gradients (but see e.g., Behangana et al., 2009), but they logically extend from patterns observed for species richness and diversity (for a review of potential causes for these patterns see McCain & Grytnes, 2010). As species approach the limits of their elevational range, reproductive capacity tends to decline, despite remarkable physiological adaptations and forms of acclimations of many high‐elevation anurans (Brattstrom, 1968; Christian, Nunez, Clos, & Diaz, 1988; Navas, Carvajalino‐Fernández, Saboyá‐Acosta, Rueda‐Solano, & Carvajalino‐Fernández, 2013). At lower temperatures and arguably under the influence of changes in rainfall and water availability, anurans tend to be developmentally, physiologically and behaviorally limited (Navas et al., 2013; Putnam & Bennett, 1981). As a result, they have shorter breeding seasons, take a relatively long time to mature (Amat & Meiri, 2017), and produce fewer (but larger) eggs (Morrison & Hero, 2003) with low abundances. In turn, this leads to potentially high vulnerability (i.e., higher local extirpation probability) of populations living at high elevations (Morrison & Hero, 2003).

We show that larger wetlands contain higher abundances of anurans and host more species, which strengthens the notion that desiccation and wetland size reduction will be detrimental to anuran survival in VNP. Not all of these species are restricted to wetland habitats, but most will likely depend on these wetlands as spawn sites and for early life stages (Roelke & Smith, 2016). More research will be needed on the nature of species‐area relationships, the natural history, and breeding micro‐habitat requirements (i.e., where the various species spawn) of anurans in VNP, if we are to understand future responses of these species to declines in wetland size through desiccation. For example, we recommend exploring whether habitat heterogeneity is higher in larger wetlands, which may drive higher species richness, or whether larger wetlands contain a larger random sample of species from the regional species pool (Lomolino, 2000).

Although we deem it probable that both elevation and wetland size play a role in observed differences among anuran communities across VNP wetlands, we acknowledge that various additional factors could also have contributed to these patterns. For one, there is a host of both local (e.g., species interactions) and landscape‐level factors (e.g., characteristics of the surrounding vegetation) that can influence differences in anuran distribution across our focal wetlands (see e.g., Van Buskirk, 2005). Isolation of populations (Scheffer et al., 2006) and stochastic processes (reviewed in Marsh & Trenham, 2001) could similarly affect dispersal, local population persistence, and local extinction, and thus shape local anuran communities.

We also acknowledge that some of our estimates of richness and diversity may be confounded due to the lack of clarity on the taxonomy and phylogeny of East African anurans (see e.g., Channing et al., 2016; Roelke et al., 2011; Zimkus et al., 2010; Zimkus & Schick, 2010). This problem is exacerbated by the uncertainty regarding the identity of at least some of the individuals we found. Thus, we emphasize that our estimates of abundance, richness, and diversity should be interpreted in the light of the spatial patterns that we explored and should not be seen as exact estimates.

Although Roelke et al. (2011) provided an overview of the anurans present in VNP, and others have addressed aspects of anuran community composition of either the larger Albertine Rift region (Plumptre et al., 2007) or a specific wetland (Lac Ngezi) in VNP (Sinsch, Greenbaum, Kusamba, & Lehr, 2011), our study is the first to provide data on anuran community composition across different wetlands in the park. Our results suggest that species composition is highly variable across wetlands (Table 3), illustrated by the fact that we were unable to detect all nine species in one single wetland. Although these differences in local community composition and relative abundances of varying species could stem from sampling biases, they also suggest that every single wetland in VNP is worth conserving. Indeed, we found that wetlands differed significantly in anuran community composition, and at least some species are likely to be found only in a few, small wetlands and are uncommon anywhere else. For example, though *Hyperolius castaneus *and *Leptopelis karissimbensi/kivuensis *were abundant in all wetlands, which is in line with previous studies (e.g., Sinsch et al., 2011), others were not as commonly recorded. Notably, *Sclerophrys kisoloensis *was only recorded in two wetlands.

Our study shows that there are at least eight anuran species in VNP. However, estimated species richness for our largest focal wetland (the approximately 45,355 m^2^ large Shegesha) shows that we potentially failed to record a few species. First, we are likely to have recorded both *L. karissimbensis *and *L. kivuensis*, but as field identification based on solely morphological features is likely to be a source of inaccuracy, we lumped them here for our analyses. Second, we have recorded both *P. graueri *and *P. parvulus* (Supporting Information Appendices S1 and S2), but although we made distinctions between the two species in the field, we lumped both species for analyses to avoid biases derived from potential misidentification. Third, Roelke and Smith (2010), Roelke and Smith (2016) proposed that both *Arthroleptis adolfifriederici* and *Xenopus wittei* could be present in VNP, whereas Sinsch et al. (2011) mentioned the presence of *Phrynobatrachus acutirostris* and *P. versicolor*, which would put the total number of species for this park at approximately fourteen. We might have failed to record these species due to incomplete sampling and acknowledge potential biases introduced by our field methodologies (e.g., not sampling at night and not sampling deeper water where *X. wittei *might be found). To more thoroughly understand patterns of anuran diversity in VNP and to get a complete record of all species present, we will need to find ways to address issues of unequal detection probability (e.g., as addressed by Sinsch et al. (2012)), and increase our sampling efforts toward a more complete sampling across seasons and hours of the day. Most anuran species show seasonal variation in activity (Goyannes‐Araújo et al., 2015; Moreira & Barreto, 1997), and not taking this into account might lead to underestimates of richness, diversity, and especially abundance. Sampling at dusk or night would similarly allow us to gather more complete data on anuran presence (Sinsch et al., 2012), but is now limited due to logistic restrictions. One possible solution to logistic limitations to nocturnal surveys would be to conduct automated acoustic sampling, which would not require our presence in the field at night (Acevedo & Villanueva‐Rivera, 2006).

The main challenge for conservation of montane anurans will be to understand the response of individual species to climate change (Beniston, 2003; Duarte et al., 2012), drying of wetlands (McMenamin et al., 2008), and a host of other threats. Certain species may be able to adapt to novel environmental conditions (Duarte et al., 2012; Hoffmann, Chown, & Clusella‐Trullas, 2013), whereas others may require more dramatic conservation actions, such as habitat restoration or translocations (Carvalho, Brito, Crespo, & Possingham, 2010; Shoo et al., 2011). A first step in setting the stage for adequate protection would be the implementation of long‐term monitoring protocols. Here, we report how such long‐term monitoring reveals spatial patterns in anuran species distribution in VNP wetlands, findings which serve as a baseline for future monitoring in a region where even basic descriptions of the natural history of most species is still lacking.

## CONFLICT OF INTEREST

None declared.

## AUTHOR CONTRIBUTIONS

DT conceived of the study, designed the study, collected the data, and helped draft the manuscript; WE contributed to the design of the study, data interpretation and manuscript writing; MAD performed data analysis, interpreted analyses, and helped draft the manuscript; NG aided with data formatting, analysis, and interpretation as well as drafting the manuscript; YvdH interpreted data analyses, and drafted the final manuscript. All authors provided comments and approved the final manuscript.

## Data Availability

Species abundances and richness data, used in this manuscript, are available in Table 3, whereas a full list of species records can be found in Supporting Information. Data available from the Dryad Digital Repository: https://doi.org/10.5061/dryad.92m95r1.
