# Supplementary material for: Spatial variation in anuran richness, diversity, and abundance across montane wetland habitat in Volcanoes National Park, Rwanda
Source: Ecol Evol. 2019 Mar 13;9(7):4220–30. doi: 10.1002/ece3.5054 (PMC6468056; doi:10.1002/ece3.5054)
Supplement: Supplementary file 2 [file ECE3-9-4220-s002.pdf]

**Appendix S2.** Photographs of all eight anuran species recorded in Volcanoes National Park, Rwanda, during our surveys.

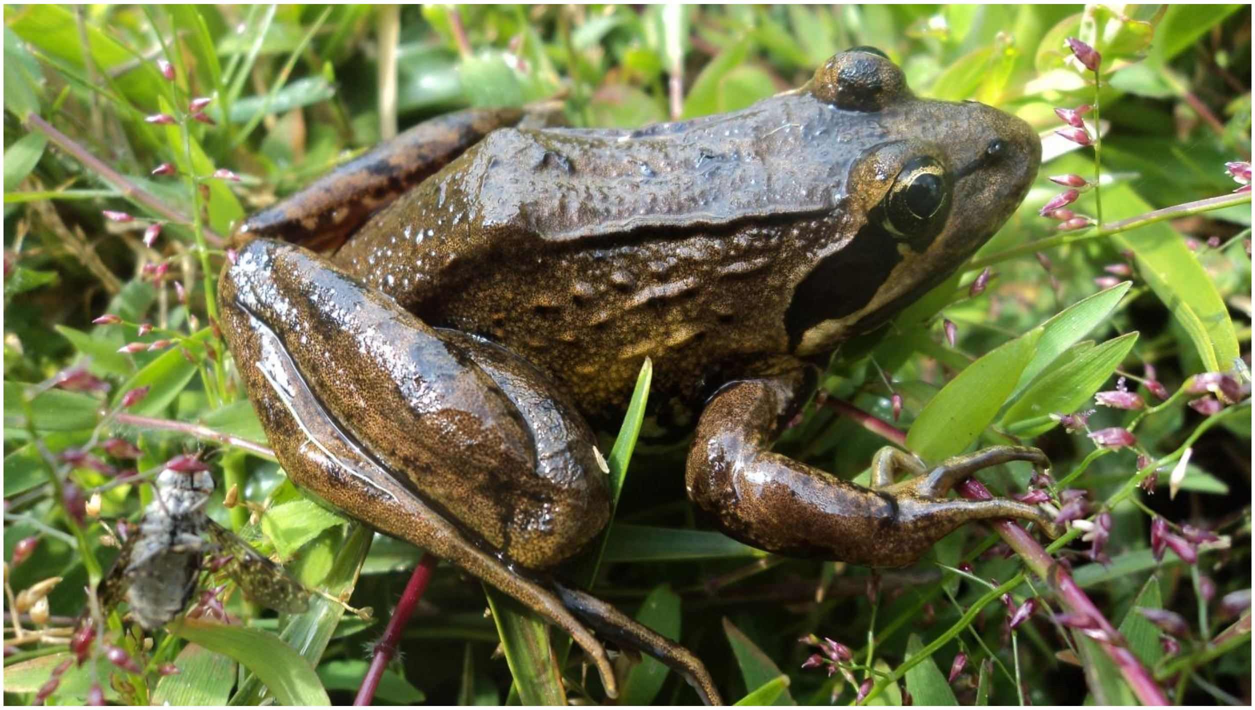

*Amietia nutti*

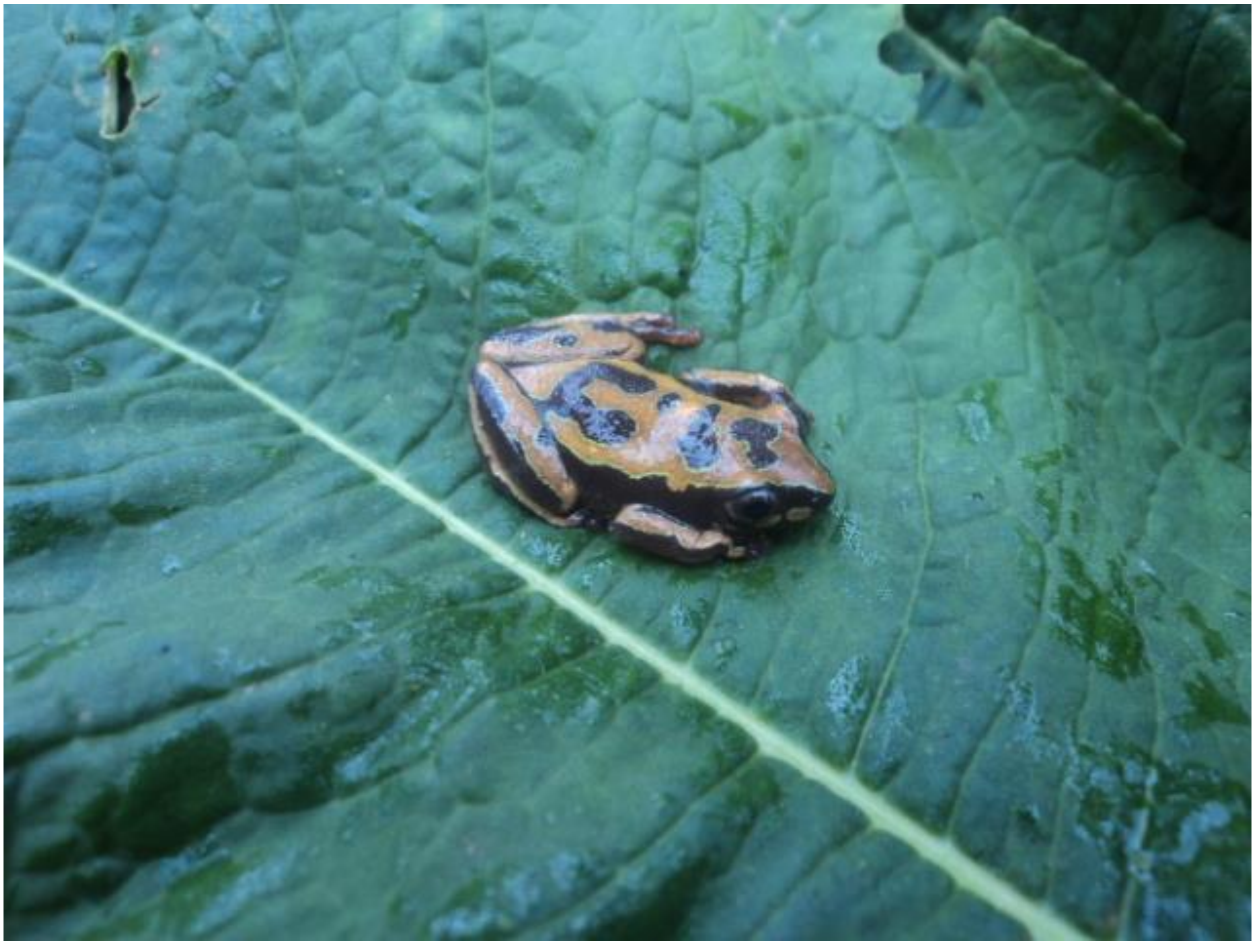
*Hyperolius castaneus*

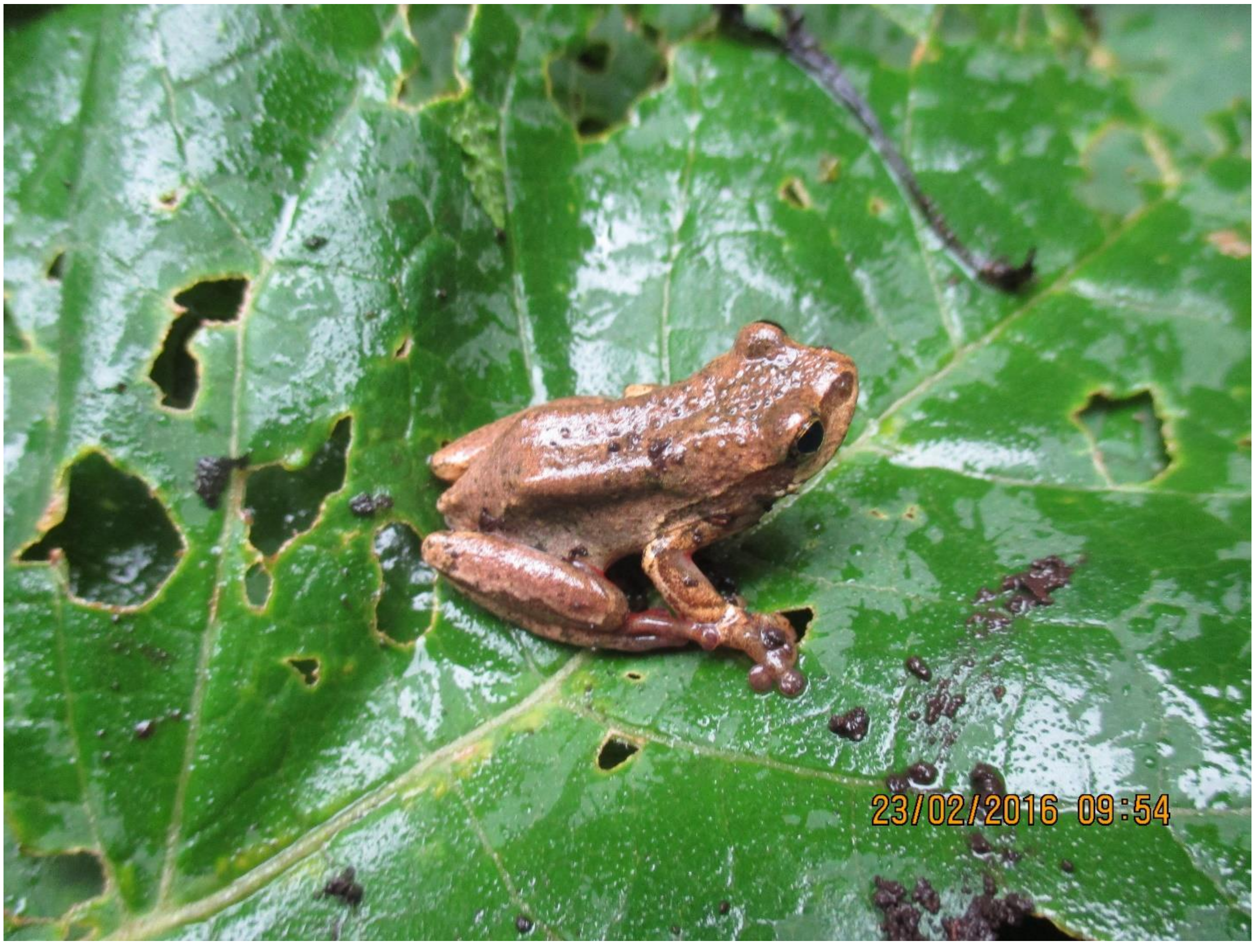

*Hyperolius cinnamomeoventris*

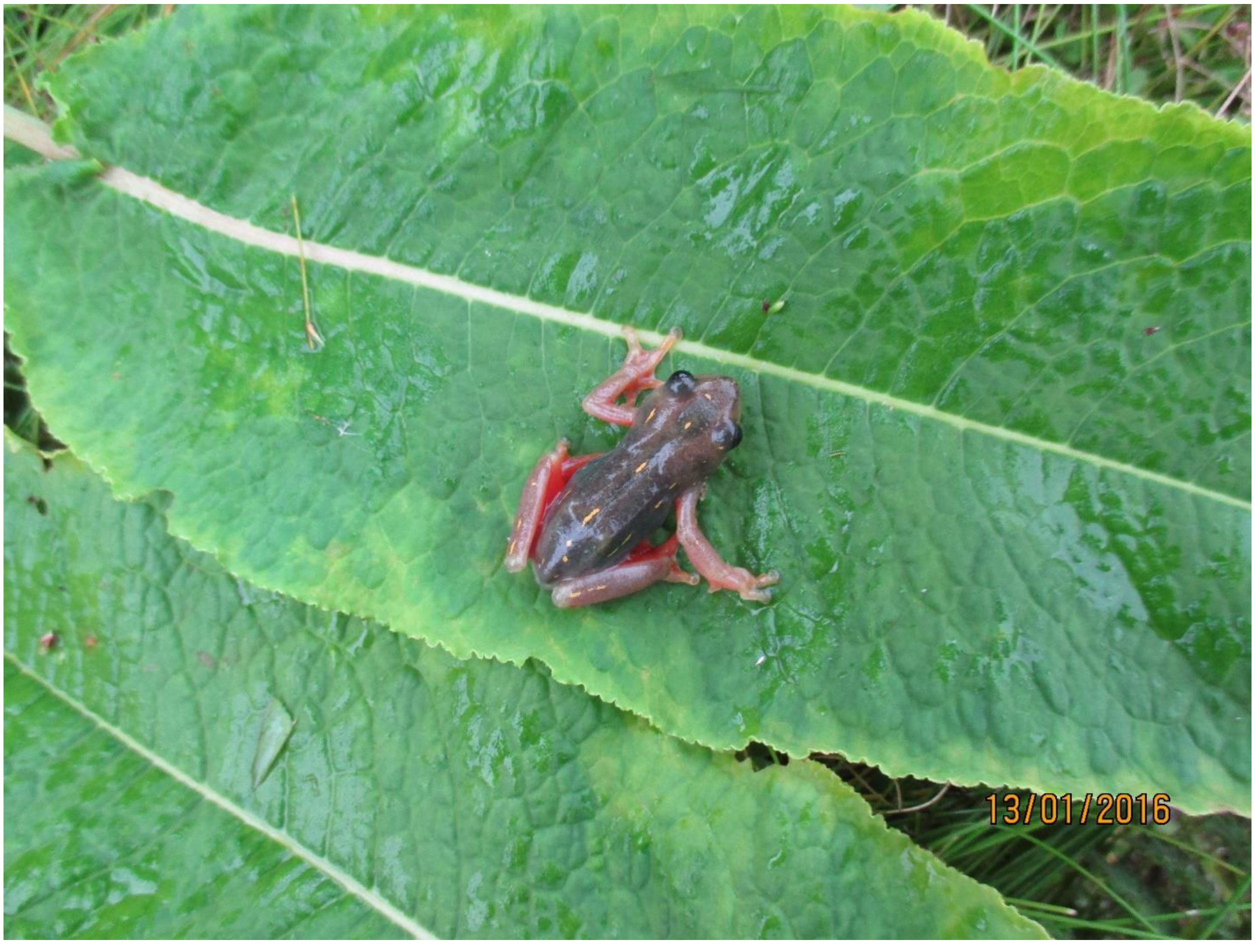


*Hyperolius viridiflavus*

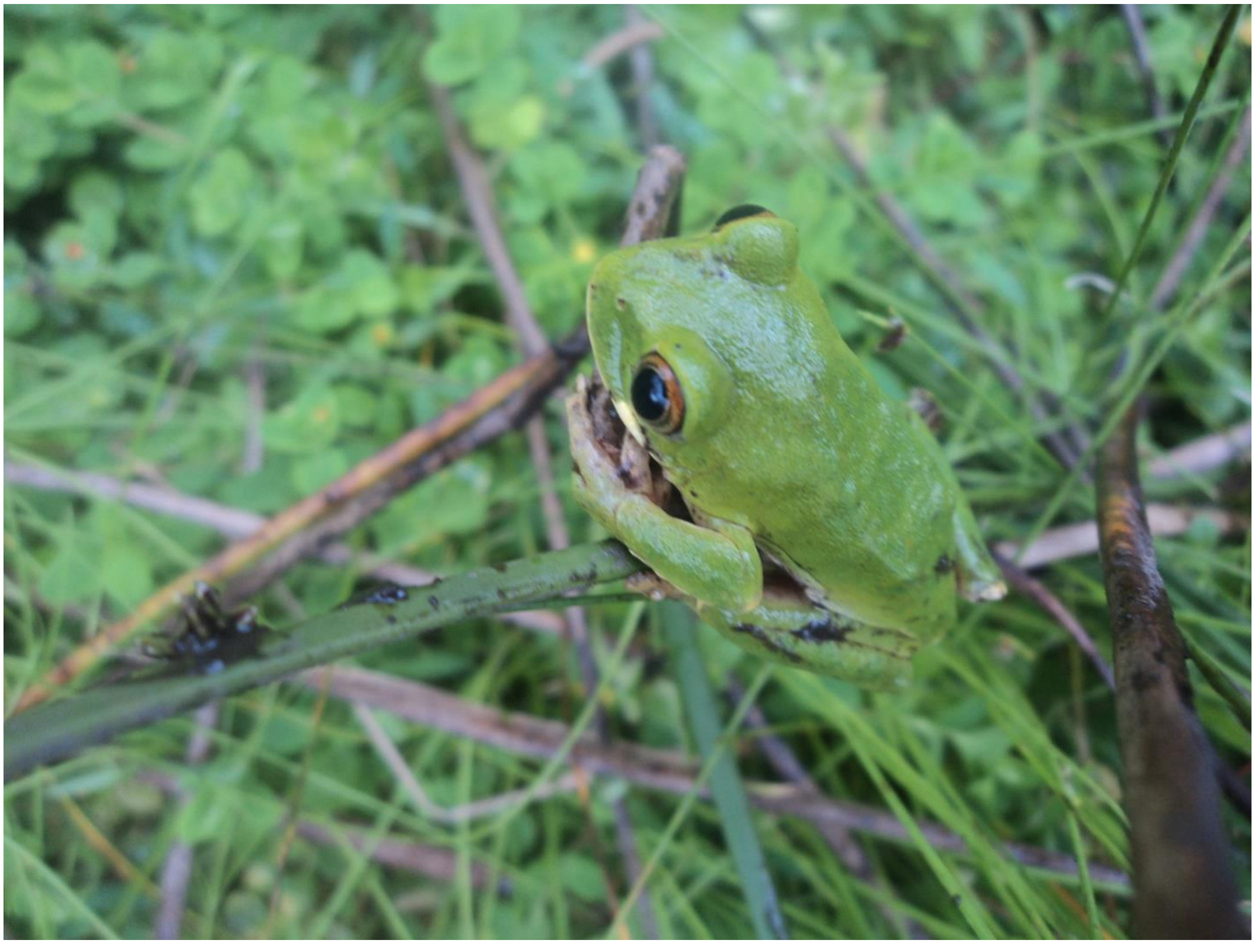

*Leptopelis karissimbensis*

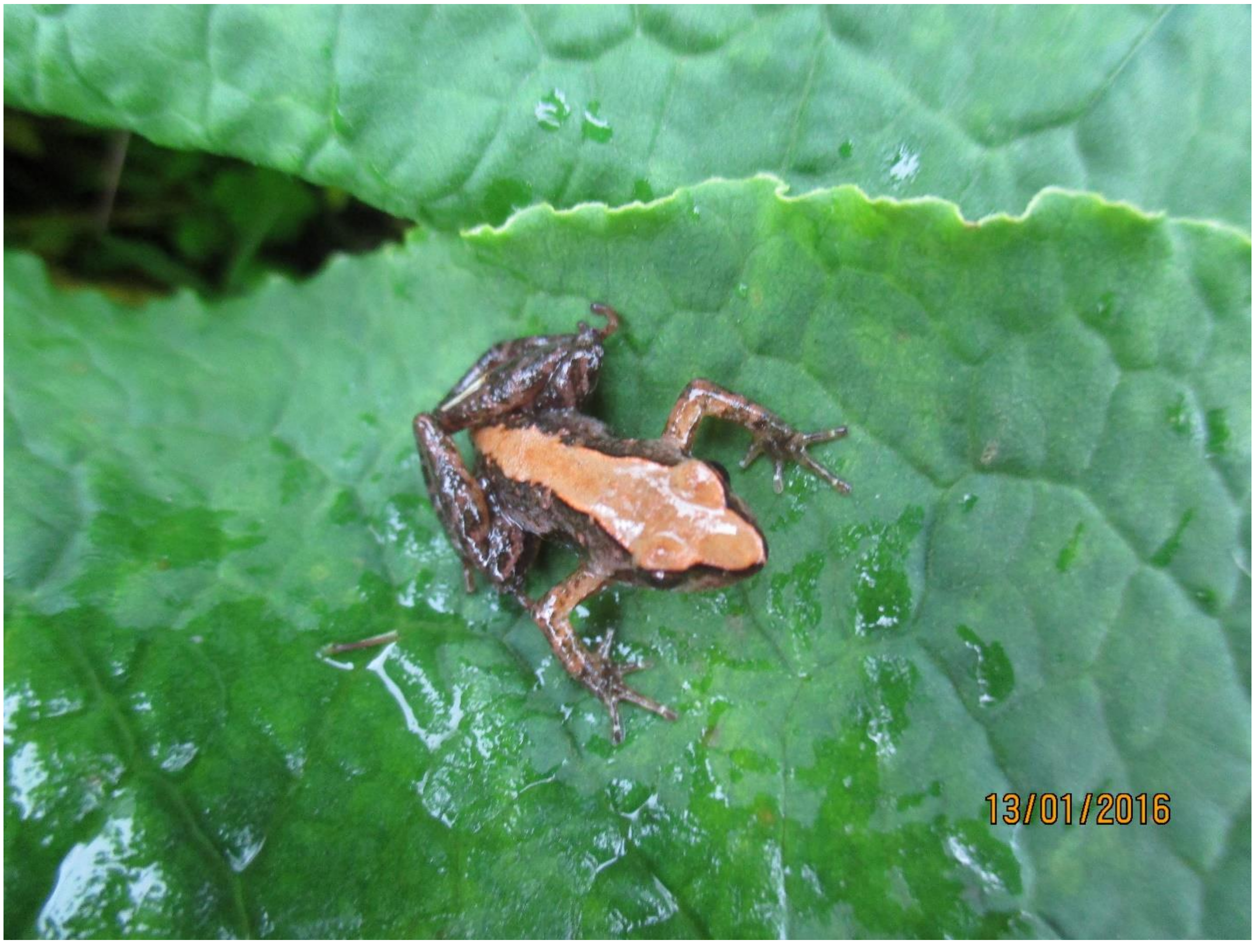


*Phrynobatrachus graueri*

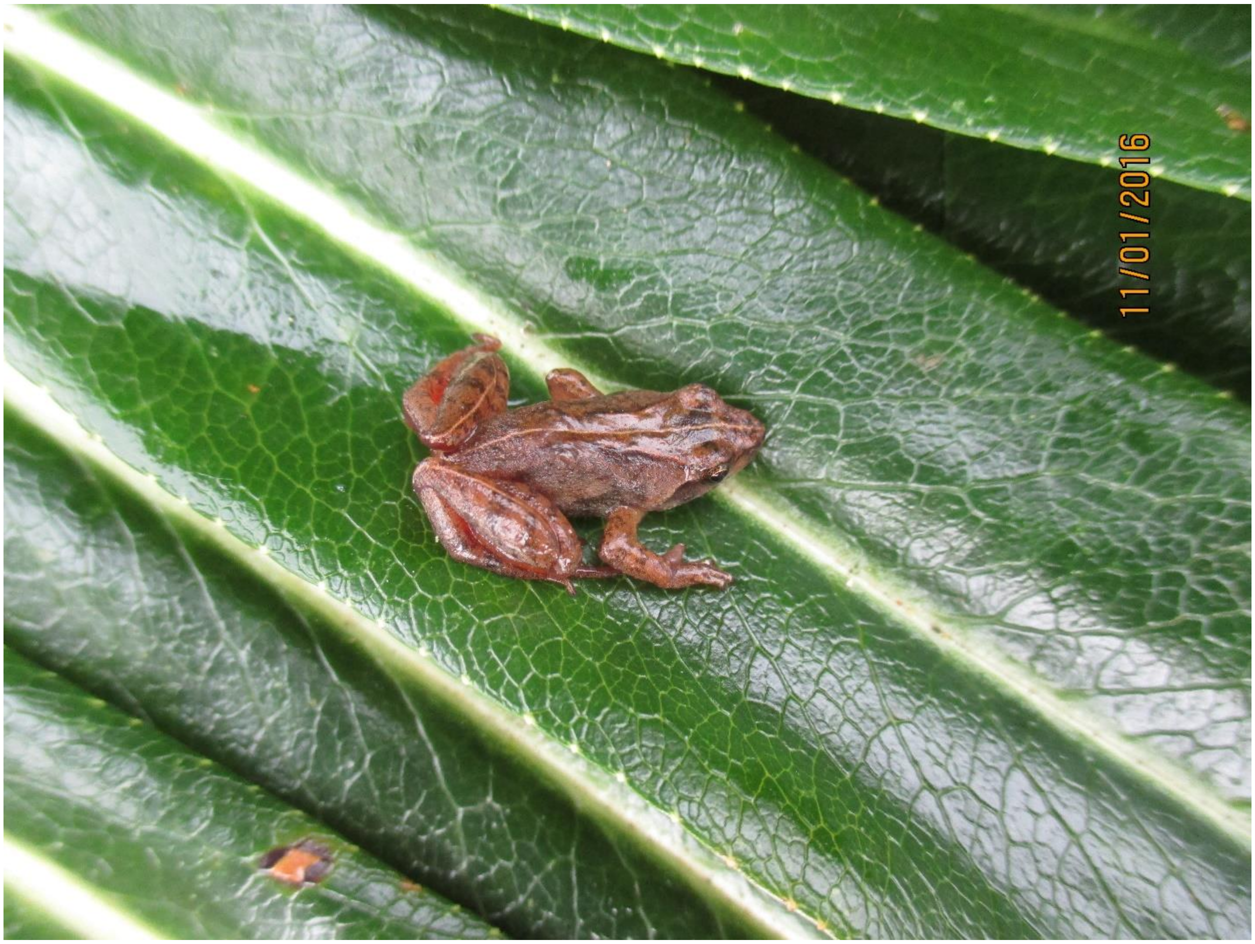


*Phrynobatrachus parvulus*

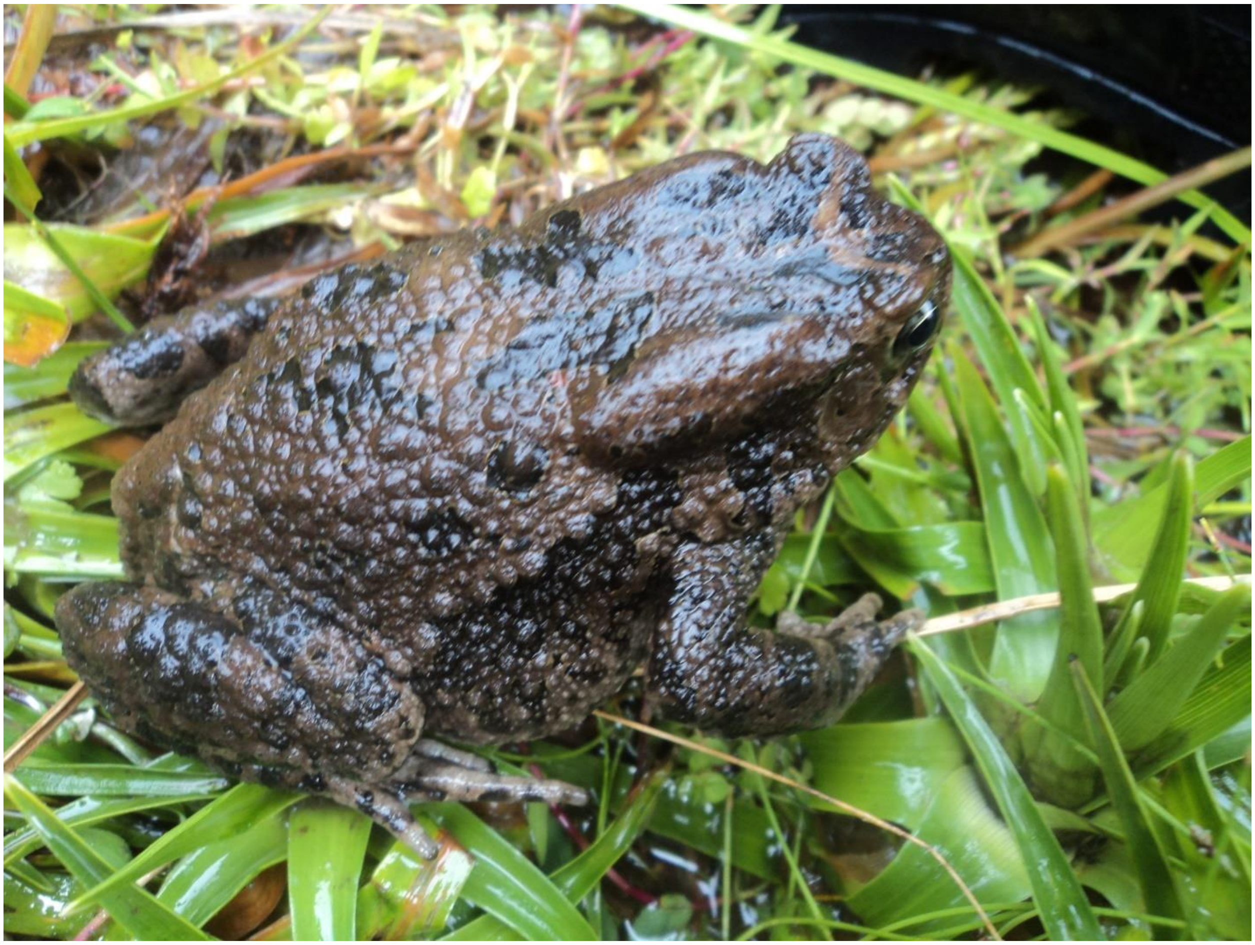

*Sclerophrys kisoloensis*
